# An efficient and cost-effective method for purification of small sized DNAs and RNAs from human urine

**DOI:** 10.1371/journal.pone.0210813

**Published:** 2019-02-05

**Authors:** Kayvan Zainabadi, Vaigundan Dhayabaran, Kutty Moideen, Patnam Krishnaswamy

**Affiliations:** 1 Genomics and Central Research Laboratory, Department of Cell Biology and Molecular Genetics, Sri Devaraj Urs Academy of Higher Education and Research, Tamaka, Kolar, India; 2 Department of Biochemistry, Indian Institute of Science, Bangalore, India; Defense Threat Reduction Agency, UNITED STATES

## Abstract

Urine holds great promise as a non-invasive sampling method for molecular diagnostics. The cell-free nucleic acids of urine however are small, labile, and difficult to purify. Here an efficient method for the purification of these nucleic acids is presented. An empirically derived protocol was devised by first identifying conditions that allowed recovery of a 100 base pair (bp) DNA, followed by optimization using a quantitative polymerase chain reaction (qPCR) assay. The resulting method efficiently purifies both small sized DNAs and RNAs from urine, which when combined with quantitative reverse transcription PCR (qRTPCR), demonstrably improves detection sensitivity. Fractionation experiments reveal that nucleic acids in urine exist both in the cell-free and cellular fraction, roughly in equal proportion. Consistent with previous studies, amplicons > 180bp show a marked loss in PCR sensitivity for cell-free nucleic acids. Finally, the lysis buffer developed here also doubles as an effective preservative, protecting against nucleic acid degradation for at least two weeks under simulated field conditions. With this method, volumes of up to 25ml of whole urine can be purified in a high-throughput and cost-effective manner. Coupled with its ability to purify both DNA and RNA, the described method may have broad applicability for improving the diagnostic utility of urine, particularly for the detection of low abundant targets.

## Introduction

Owing to its non-invasive nature, the use of urine for detecting human pathogens (via amplification of pathogen-associated nucleic acids with polymerase chain reaction, PCR) is gaining traction as a useful diagnostic tool [1–10]. This utility was highlighted during the recent Zika and Ebola outbreaks where PCR testing of urine was found to be a highly effective strategy for identifying infected individuals [11–17].

In the case of Zika and Ebola, infection of renal proximal tubular epithelial cells leads to shedding of virus particles into the urine [15–17]. However, the infectious agent itself need not necessarily be present in urine for detection of disease. For instance, both tuberculosis (TB) and human papillomavirus (HPV) infection have been successfully diagnosed with PCR testing of urine samples [18–21]. In fact, a recent meta-analysis demonstrated that urine-based diagnosis of high-risk HPV infection (a significant risk factor for cervical cancer) had a sensitivity of 77% and specificity of 88% as compared to traditional pap tests [18]. For TB, the results have been more variable: compared to traditional sputum based microscopy/culturing methods, urine has had a sensitivity ranging from 7–100% [19–21]. While encouraging, the sensitivity and specificity of these methods are still too low and inconsistent to justify their use for routine screening, highlighting the need for further improvements.

Exactly how certain pathogenic DNAs make their way into the urine is still an open question. While it is easier to imagine HPV infected urogenital cells (or mucosal secretions containing virus particles) shedding into the urine, it is harder to explain the presence of pulmonary tuberculosis. Since TB normally cannot be cultured from urine [22, 23], it is more likely that TB cell-free DNA is being detected in urine. These extracellular nucleic acids are thought to be first released into the serum from dying cells and then eventually make their way into the urine after filtration through the kidneys (upon degradation to less than a few hundred base pairs) [24, 25]. A second parallel pathway may involve secretion through exosomes (small lipid enclosed vesicles shed by cells) which have been shown to harbor nucleic acids and be present in urine [26].

Urine contains a mixture of both cell-free and cellular nucleic acids. While the latter are large in size and correspond predominantly to that of the host (i.e. from the epithelial lining of the urogenital tract), cell-free DNAs are small and enriched for nucleic acids from distant parts of the body, including pathogens and/or disease states [27, 28]. The abundance of cell-free nucleic acids in urine may depend on a number of key variables, including the overall health of the individual, the disease state being evaluated, and the sampling, extraction and detection strategy being employed [25, 27–30]. For instance, the first-void fraction of urine has been shown to contain a much higher concentration of total nucleic acids as compared to mid-stream or other fractions [30]. Importantly, the small size and extracellular nature of cell-free nucleic acids make them labile and difficult to purify with standard methods.

While cell-free nucleic acids show great diagnostic potential, the molecular tools necessary for harnessing their full potential are currently limited. Only a handful of studies have systematically evaluated how key variables such as sample collection, storage, and nucleic acid purification/amplification strategies affect the performance of urine-based diagnostic tests [29–33]. Consequently, many disparate protocols have arisen, leading to varying and sometimes conflicting results [18–21]. Further, the lack of standardized protocols has led to a somewhat over-reliance on kit-based purification strategies (i.e. Qiagen QIAamp), which while providing a simple and standardized way to purify DNA, have the stated inability to purify DNAs ≤ 100bp (and reduced efficiency for DNAs < 200bp) [34–36], the size of most cell-free DNAs. These kits are also prohibitively expensive for routine use in many parts of the world and are typically compatible with only small volumes (< 1ml) of urine.

Here an efficient, cost-effective, and high-throughput method for purifying small sized nucleic acids from large volumes of urine was devised. Intriguingly, the method was found to purify both DNA and RNA, which in conjunction with reverse-transcription PCR markedly improved detection sensitivity. These findings may have implications for improving the use of urine for molecular diagnostics.

## Materials and methods

### Urine collection, storage and processing

This study was reviewed and approved by the Human Subjects Ethical Review Board at Sri Devaraj Urs Academy of Higher Education and Research. De-identified urine samples (the first 50ml of the urine stream) were collected in the morning from healthy volunteers at Sri Devaraj Urs Medical College and used same-day for all experiments. This fraction of urine has previously been shown to contain the greatest concentration of total nucleic acids as compared to other fractions [30]. If not immediately used, samples were kept at 4°C and then allowed to re-equilibrate to room temperature prior to use. Whole urine was used for all experiments (unless otherwise indicated), with samples mixed thoroughly prior to use in order to resuspend pelleted material. While the use of daily fresh urine led to variability in absolute qPCR cycle threshold (Ct) values from day-to-day experiments, only relative differences between different conditions were evaluated, thus de-emphasizing absolute Ct values.

For optimization experiments, de-identified urine samples were obtained from healthy male volunteers and then pooled. Once the protocol was finalized, de-identified urine from healthy female volunteers was also analyzed as a control. For cell-free nucleic acid experiments, whole urine was centrifuged in an Eppendorf Centrifuge 5424 (rotor FA-45-24-11) at either 7,500 RPM (5,283 g) or 15,000 RPM (21,130 g) for 10 minutes at 4°C. The resulting supernatant and pellet (and whole urine stored at 4°C) were extracted and analyzed at the same time. For stability studies, fresh urine was aliquoted (after mixing) into different tubes containing various preservatives and stored for up to 2 weeks at 28°C to simulate field conditions. Identical samples stored at 4°C, -20°C, and -80°C (all without preservative) served as controls.

### Extraction conditions

A guanidine (chaotropic salt) and silica purification strategy was chosen as a starting point for optimizing extraction conditions due to its: 1) Powerful ability to purify nucleic acids from even complex mixtures; 2) High denaturing ability which helps protect nucleic acids from DNase and RNase-mediated degradation; and 3) Cost-effectiveness [37]. The final working protocol is presented in **S1 Appendix** and a list of all consumables and equipment is presented in **S1–S3 Tables**.

Initial tests focused on urine samples spiked with a 100bp ladder in order to monitor the ability of different lysis/wash conditions to purify small DNAs that are associated with cell-free nucleic acids. In these experiments, lysis buffer was mixed with urine at a ratio of 2:1; Wash 1 consisted of the lysis buffer diluted 2:1 with water; and Wash 2 consisted of 70% ethanol. After purification, eluates were separated on a 2% agarose gel and compared to a lane containing an equivalent amount of 100bp ladder. Conditions that enriched for retention of the 100bp band were then tested for their ability to purify total nucleic acids using a quantitative polymerase chain reaction (described below).

Initially, a 2:1 volume of lysis buffer to urine was used (500μl lysis buffer + 250μl urine), but subsequently changed to a 1:1 volume (375μl lysis buffer + 375μl urine) to allow for purification of larger sample volumes. For these latter experiments, urine was thoroughly mixed with lysis buffer (containing appropriate chaotropic agent, 20mM Tris HCl pH 7.4, 50mM EDTA, 4% Triton-X100) with or without alcohol, with Nunc 96 Deep Well DNA filter plates (Z688673, Sigma-Aldrich) used for total volumes less than 800μl. 750μl of the urine/lysis buffer mixture was loaded into a well of the DNA plate, centrifuged using Eppendorf Centrifuge 5810 (rotor A-2-DP) at 3,700 RPM (2,250 g) for 1 minute, washed with 500μl of Wash 1 (lysis buffer without 2-mercaptoethanol diluted 1:1 with water), spun at 3,700 RPM (2,250 g) for 1 minute, washed with 500μl of Wash 2 (25% ethanol, 25% isopropanol, 100mM NaCl, 10mM Tris pH 7.4), spun at 3,700 RPM (2,250 g) for 2 minutes, dried in a 56˗65°C incubator for 10 minutes, eluted with 50μl of TE (Tris EDTA) buffer pH 8.0 and spun at 3,700 RPM (2,250 g) for 2 minutes. Please note that guanidine containing lysis and Wash 1 buffers are hazardous and incompatible with bleach.

For larger urine volumes, a similar protocol was followed except that silicon dioxide binding solution was added directly to the urine/lysis mixture (please see protocols **S2–S4 Appendices** and schematic **S5 Appendix**). Silicon dioxide (S5631, Sigma Aldrich) consists of particles 0.5–10μm in size; in order to preferentially retain the larger silica particles, 2.5 grams of silicon dioxide was thoroughly mixed with 50ml of water in a 50ml tube, allowed to sit for 2 hours, and the supernatant (consisting of small silica particles) discarded. This procedure was repeated 2 additional times and the resulting pellet was resuspended with 20ml of lysis buffer (herein referred to as binding solution). Before use, the binding solution was thoroughly vortexed to resuspend the silica particles and 150μl, 250μl, or 500μl of binding solution was added to final urine/lysis mixtures of 5ml, 15ml, or 50ml, respectively. After addition of binding solution to the urine/lysis mixture, the resulting solution was thoroughly mixed, then centrifuged at 3,000 RPM (1,640 g) for 1 minute, the supernatant discarded, and the resulting silica pellet resuspended with 500μl of Wash 1 and transferred to a well of a Pall GHP .45μm 96-well size exclusion plate (89233–870, VWR). The remainder of the protocol is identical as described above starting from Wash 1.

### Quantitative polymerase chain reaction (qPCR)

A qPCR approach was utilized to quantitatively assess the yield and purity of nucleic acids from urine. As urine contains a mixture of both cellular and degraded/cell-free DNA, the shortest possible amplicon for the human *actin* housekeeping gene was chosen so as to prevent any bias based on the size or source of the nucleic acids. To test the effect of amplicon size, the probe and reverse primer for *actin* were kept constant, with only the forward primer changed to yield increasingly larger amplicon sizes (**S6 Appendix**). Primer length, GC content and other variables were kept as constant as possible to minimize PCR efficiency differences. None of the primer sets spanned an intron, thereby allowing amplification of both DNA and RNA (after reverse-transcription, RT). In experiments comparing qRTPCR (which targets both RNA and DNA) to traditional qPCR (which only targets DNA), identical QuantiTect multiplex RTPCR master-mix (Qiagen) containing both Taq DNA polymerase and RT enzyme (Qiagen) was used, except in the case of qRTPCR an initial step 50°C for 20 minutes was added prior to PCR amplification to allow for reverse transcription (**S6 Appendix**). For DNA-based qPCR this first step was skipped. Samples were run on the same day, at the same time, and on identical LC96 machines (Roche) as previously described [38]. The data presented represents the average of at least two biological replicates and two technical replicates performed at least two independent times.

## Results

### Optimizing extraction conditions

**Fig 1:**
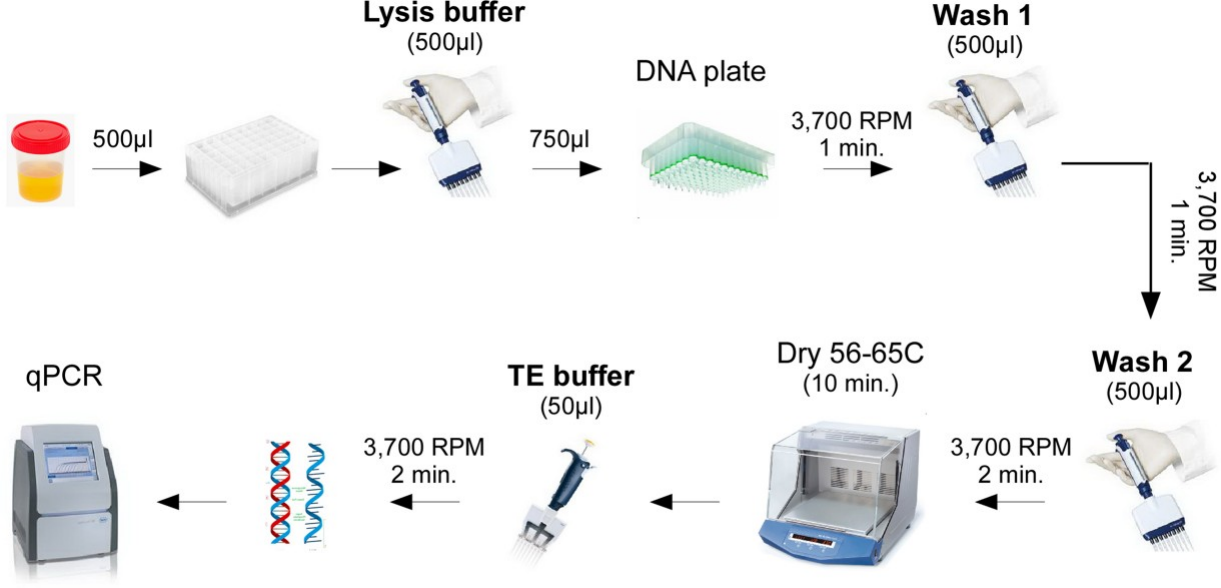
A schematic representation of the protocol. The complete protocol can be found in S1 Appendix and a list of consumables in S1 Table.

**Fig 2:**
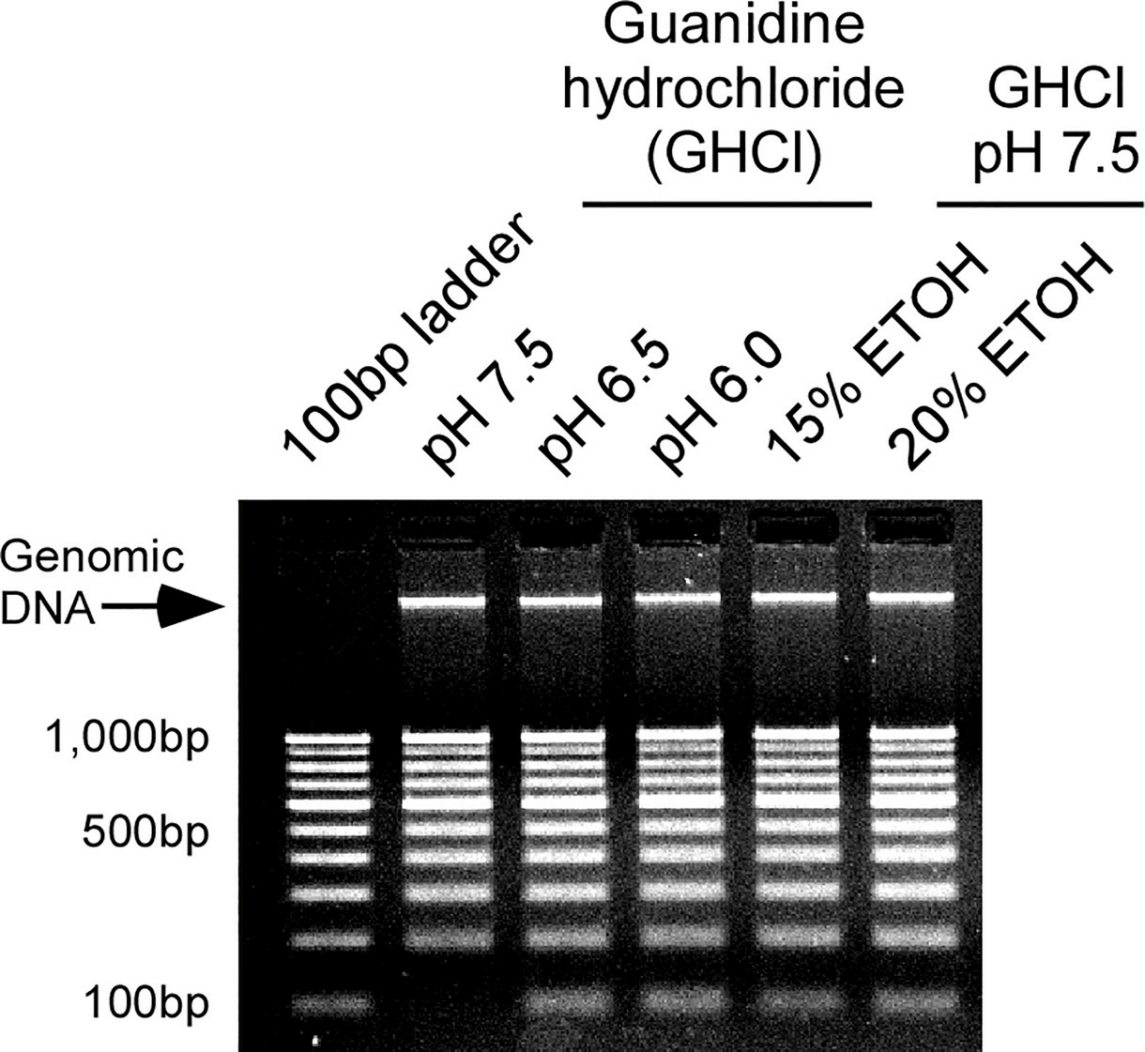
Optimization for recovery of small DNAs from urine. Human urine was spiked with a 100bp DNA ladder and then extracted using different conditions. An equivalent amount of ladder was run in the first lane for comparison. Only lysis buffers with lowered pH (6.5–6.0) or which contained alcohol (ETOH, ethanol) efficiently recovered the 100bp band.

Many commercial kit based methods have the stated inability (or reduced efficiency) for purifying the small sized nucleic acids that are typical of cell-free DNAs [34–36]. Such kits are also oftentimes too expensive for routine use in many parts of the world. As a result, an attempt was made to devise a more cost-effective method for purifying cell-free nucleic acids (**Fig 1**). To achieve this, a number of different extraction conditions were first tested using urine spiked with a 100bp DNA ladder. As **Fig 2** shows, while all conditions were able to purify high molecular weight genomic DNA from urine, only lysis buffers with lowered pH or which contained alcohol were able to purify the smallest 100bp band of the DNA ladder.

Since total nucleic acid yield does not account for the co-purification of contaminants (such as PCR inhibitors which urine is known to contain [39]), a quantitative PCR (qPCR) strategy was next taken to further optimize the protocol. A 60bp product of the human housekeeping *actin* gene was chosen as the target since its widespread expression and small amplicon size would minimize any bias based on the size or source of the purified nucleic acids (**S6 Appendix**). A number of different extraction variables were then tested for their effect on qPCR cycle threshold (Ct) values, with a lower Ct value corresponding to an improvement (**S7–S10 Appendices**). Two of the key findings from these experiments are: 1) The inclusion of alcohol in the lysis buffer markedly improved Ct values when a reverse-transcription step was incorporated prior to qPCR (by up to 4 values), indicating co-purification of RNA (**Table 1**); and 2) Use of a stronger chaotropic salt (guanidine thiocyanate, GuSCN) and higher isopropanol concentration (33%) improved overall nucleic acid recovery (**Table 2, S7 Appendix)**. The resulting final working protocol (**Fig 1, S1 Appendix)** uses 3M GuSCN with 33.3% isopropanol (at a 1:1 ratio of lysis buffer to urine) for extraction, and qRTPCR for detection in order to account for total nucleic acids. One interesting tangential finding from these experiments is that addition of 1mg/ml bovine serum albumin (BSA) directly to urine samples (but not to the lysis or wash buffers) led to a consistent improvement in qRTPCR Ct values (**S9 and S11 Appendices**).

**Table 1:** Addition of alcohol markedly improves the recovery of RNA from urine as assessed by qRTPCR for human actin.

	GHCl pH 6.5					
Alcohol:	-		20% ETOH		20% ISOH	
	- RT	+ RT	- RT	+ RT	- RT	+ RT
Average Ct (± SD)	31.5 (±.4)	30.9 (±.3)	32.0 (±.3)	28.5 (±.1)	31.9 (±.1)	27.9 (±.1)
ΔCt	0.6		3.5		4.0	

2:1 urine to lysis buffer; GHCl, guanidine hydrochloride; RT, reverse-transcription; Ct, cycle threshold; SD, standard deviation; ETOH, ethanol; ISOH, isopropanol

**Table 2:** Use of guanidine thiocyanate outperforms guanidine hydrochloride for purification of nucleic acids from urine as assessed by qRTPCR for human actin.

Chaotropic salt:	3M GHCl + 33% ISOH	3M GuSCN + 33% ISOH
Average Ct	32.4	30.0
(± SD)	(±.4)	(±.1)

1:1 urine to lysis buffer; GHCl, guanidine hydrochloride; GuSCN, guanidine thiocyanate; Ct, cycle threshold; SD, standard deviation; ISOH, isopropanol

### Adapting for use with larger urine volumes

In addition to its non-invasive nature, one of the advantages of urine is that large volumes can be easily collected for testing. This increased volume has the potential to compensate for targets that may occur in low abundance [28]. However, kit-based methods are either incompatible or prohibitively expensive for use with such high volumes. Consequently, the current high-throughput method, which was initially developed for use with small urine volumes, was adapted for use with larger volumes (**S10 and S11 Appendices**).

**Fig 3:**
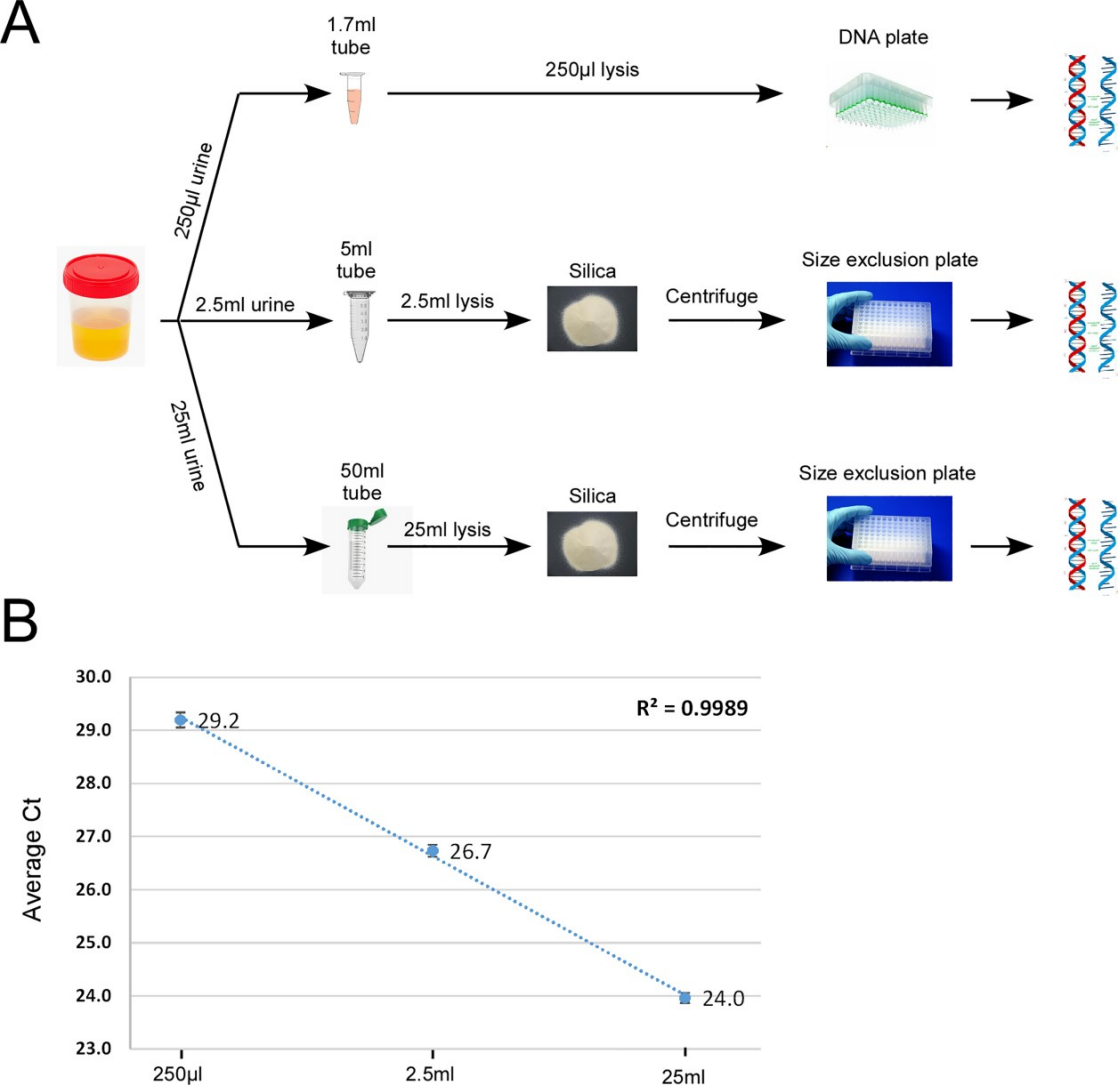
Adapting the current protocol for use with larger urine volumes. **A)** Schematic showing parallel extraction methods for use with 2.5ml and 25ml of urine. A more detailed schematic is presented in S5 Appendix and detailed protocols in S2–S4 Appendices. B) Use of larger volumes leads to a linear improvement in nucleic acid recovery as assessed by qRTPCR cycle threshold (Ct) values for human *actin*.

The final optimized protocol was tested to determine how increased sample volume affects nucleic acid recovery (**Fig 3A, S2–S5 Appendices**). As **Fig 3B** shows, increasing the volume 10-fold and 100-fold (from 250μl to 2.5ml and 25ml) improved *actin* Ct values by 2.5 and 5.2 Ct values, respectively. This linear improvement indicates that volumes of up to 25ml can be used to improve the detection of even low abundant targets.

### Confirming the presence of cell-free nucleic acids

The current method is able to purify a substantial portion of nucleic acids from urine. To determine where these nucleic acids are coming from (i.e. cellular versus cell-free), whole urine was first centrifuged at 7,500 RPM (5,283 g) or 15,000 RPM (21,130 g) to pellet cellular material, and the resulting pellet and supernatant examined for the presence of nucleic acids via qRTPCR. As expected, whole urine yielded the lowest overall qRTPCR Ct values (indicating the greatest concentration of nucleic acids), whereas the supernatant and pellet after centrifugation both showed higher, albeit similar Ct values as one another (**Table 3**). These data suggest that the cellular and non-cellular fraction of urine contribute roughly equivalent amounts of nucleic acids to that present in whole urine, at least with respect to the human target *actin*.

**Table 3:** The cell-free fraction of urine (corresponding to the supernatant) contains cell-free nucleic acids as assessed by qRTPCR for human actin.

	Whole urine	7,500 RPM pellet	7,500 RPM supernatant	15,000 RPM pellet	15,000 RPM supernatant
Average Ct (± SD)	28.8 (±.4)	30.6 (±.3)	30.3 (±.1)	31.0 (±.3)	30.9 (±.3)

Ct, cycle threshold; SD, standard deviation

### Small amplicon size improves detection of cell-free nucleic acids

**Fig 4:**
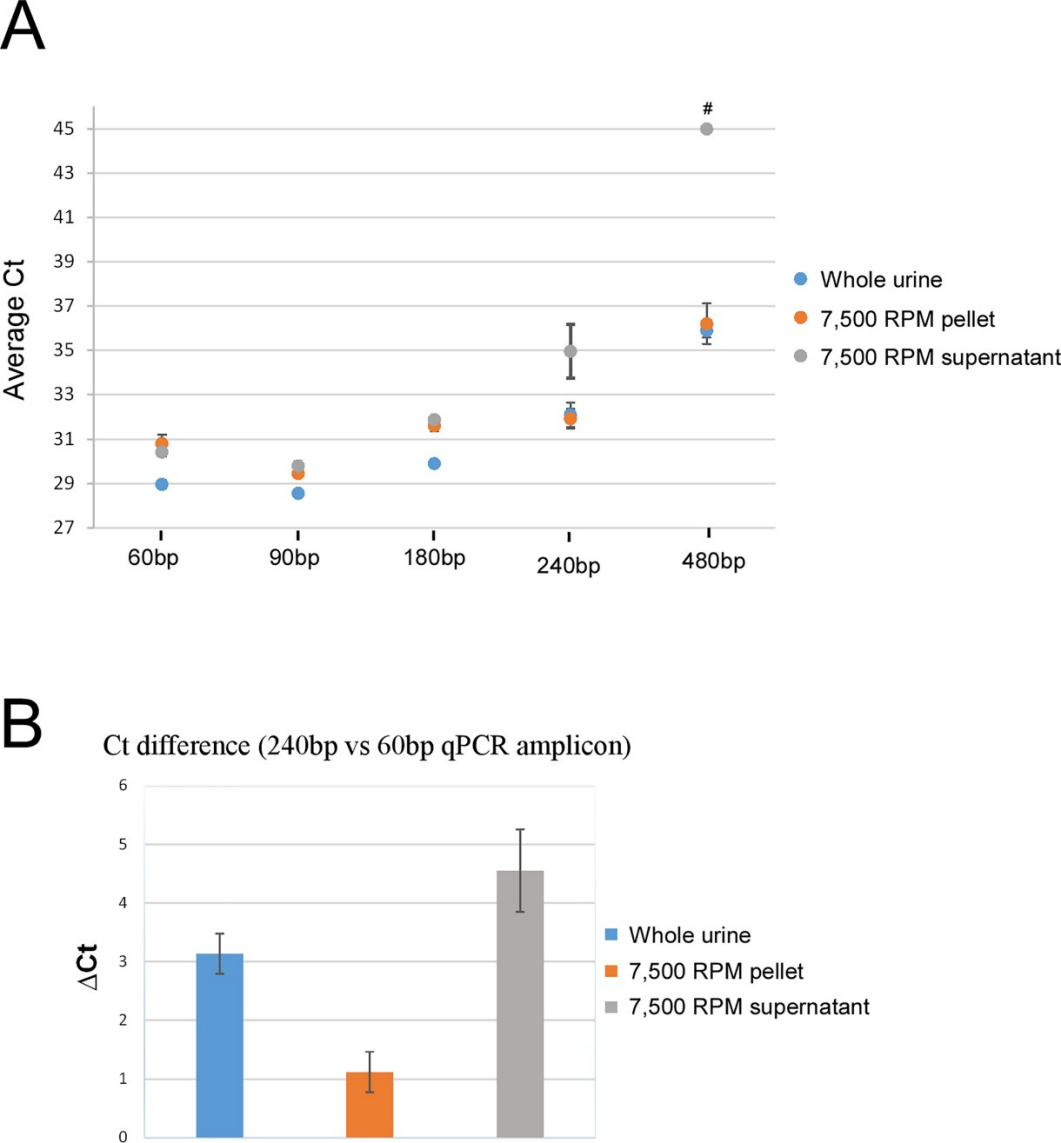
Cell-free nucleic acids are only a few hundred base pairs long. **A)** qRTPCR for human *actin* using increasingly larger amplicon size shows a loss in signal for the cell-free fraction (7,500 RPM supernatant) for amplicons > 180bp. ^#^ Undetectable. B) Comparison of Ct values between the 240bp and 60bp amplicon shows that there is a four-fold higher loss in signal for the cell-free (7,500 RPM supernatant) than cellular fraction (7,500 RPM pellet).

It is thought that the cell-free nucleic acids present in urine are generally small in nature [25–27]. To indirectly measure their size, urine was centrifuged as before and the resulting supernatant and pellet analyzed with qRTPCR using increasingly larger *actin* amplicon sizes (**S6 Appendix**). **Fig 4A** shows that for amplicons ≤ 180bp, nearly identical Ct values were obtained for the cell-free and cellular fractions. In contrast, the 240bp amplicon showed a marked loss in signal for the cell-free fraction (**Fig 4A and 4B**), which became even more dramatic for the 480bp amplicon. In both of these cases, the cellular fraction yielded near identical Ct values as whole urine, indicating that for amplicons ≥ 240bp nearly all of the qRTPCR signal was coming from the cellular component. These data confirm previous findings that cell-free nucleic acids are small in nature and underscore the importance of using small amplicon sizes (< 180bp) when attempting to amplify cell-free nucleic acids.

### Lysis buffer also acts as a preservative

The use of urine as a non-invasive sampling strategy may also be useful for epidemiological research. However, a current obstacle is that samples collected in the field oftentimes do not have access to a cold-chain. As a result, samples are subject to ambient temperatures which may negatively impact their quality and suitability for molecular testing. This is particularly cogent for urine, as cell-free nucleic acids are small and extracellular, and thus particularly susceptible to degradation. The ability to collect samples without the need for a cold-chain would greatly facilitate the use of urine for molecular epidemiology.

To determine the effect of sample storage on nucleic acid stability, whole urine was stored in simulated field conditions (28°C) for one week with and without the use of different preservatives. **Table 4** shows that the guanidine thiocyanate (GuSCN) lysis buffer used in this study showed the greatest overall protection, helping to preserve nucleic acids in urine better than established preservation methods such as EDTA, sodium azide, or even refrigeration (4°C). Remarkably, samples stored at 28°C with the GuSCN lysis buffer showed Ct values similar to or better than control samples stored at -20°C or -80°C (without preservative).

**Table 4:** One week stability study examining the effects of different preservatives on human urine.

Temperature:	28°C				4°C	-20°C	-80°C
Preservative:	-	25mM EDTA	25mM EDTA + 0.1% NaN_3_	3M GuSCN + 33% ISOH	-	-	-
Average Ct (± SD)	35.3 (±.3)	32.8 (±.2)	32.0 (±.1)	30.6 (±.1)	33.7 (±.8)	31.7 (±.5)	31.3 (±.3)

All samples were extracted with the current method.

GuSCN, guanidine thiocyanate; EDTA, Ethylenediaminetetraacetic acid; NaN_3_, sodium azide; ISOH, isopropanol; SD, standard deviation; Ct, cycle threshold

**Fig 5:**
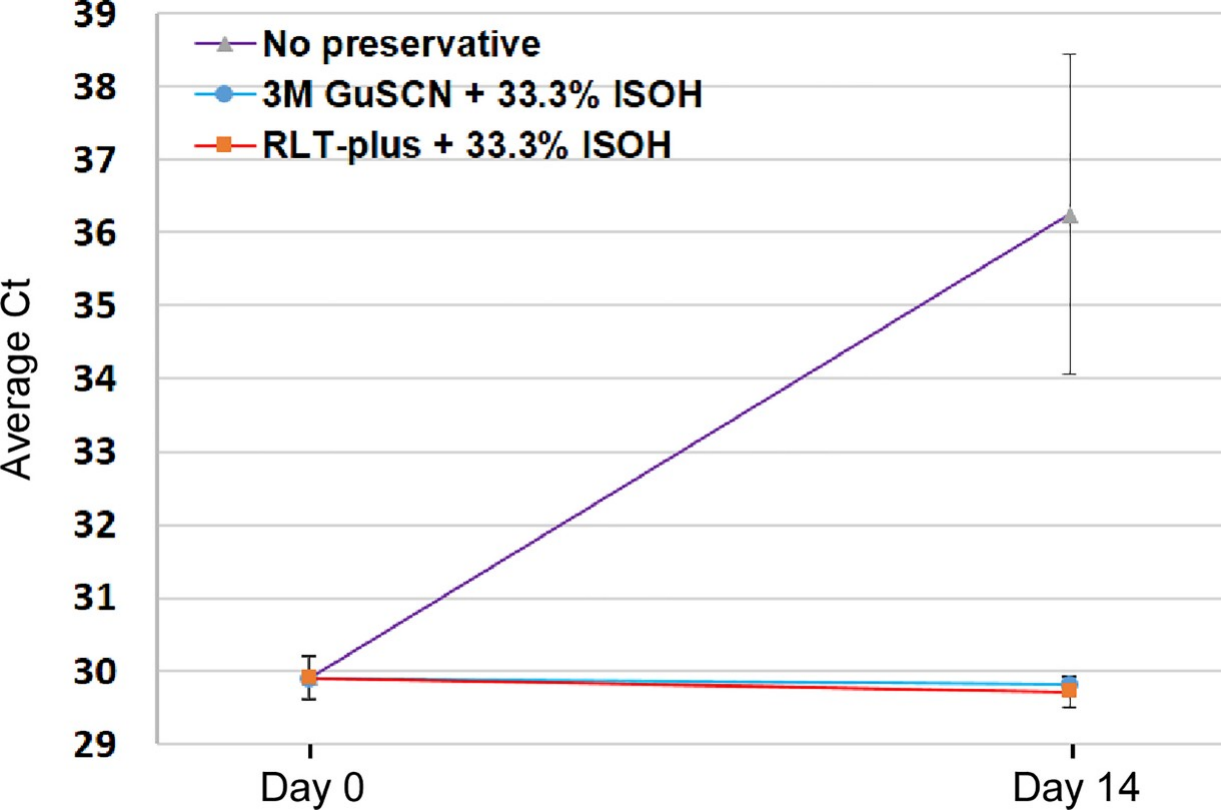
Lysis buffers also double as effective preservatives. Aliquots of human urine were stored with the indicated lysis buffers or no preservative for two weeks at 28°C. The home-made and commercial lysis buffers completely prevented any loss in Ct values as assessed by qRTPCR for human *actin*.

Finally, it has recently been reported that Qiagen RLT-plus can serve as a substitute for a similar homemade guanidine based lysis buffer [40]. RLT-plus with 33.3% isopropanol was thus tested and found to perform equivalently as the GuSCN lysis solution (**S12 Appendix**). Testing of these two buffers in a longer two week stability study showed that they both completely prevented the 6.3 Ct value loss observed for samples stored without preservative (**Fig 5**). These data indicate that urine can be stored in these lysis buffers for at least two weeks with no detectable loss in qPCR detection sensitivity.

## Discussion

Urine has the potential to serve as a non-invasive sampling method for the diagnosis of human disease and for molecular epidemiology. Numerous studies over the last decade have evaluated the diagnostic utility of urine with sometimes inconsistent results [18–21, 41–50]. This is in part due to the lack of standardized techniques for collecting, preserving, purifying and amplifying nucleic acids from urine. Additionally, the over-reliance on the use of kit-based purification strategies, which preferentially retain large DNAs at the expense of small ones, has likely hampered the field. Therefore, new methods are currently needed.

Here we present an optimized method for the purification of small sized nucleic acids from urine. The current method has the following advantages: 1) It has the demonstrated ability to purify nucleic acids as small as 100bp, a relevant property given the small size of cell-free nucleic acids; 2) It purifies both DNA and RNA, which when coupled with RTPCR demonstrably improves PCR detection sensitivity; 3) The lysis buffers developed in this study also act as preservatives, obviating the need for a cold-chain for at least two weeks; 4) The method is high-throughput and approximately three times less expensive than commercial kit-based protocols, enabling its use in the developing world or for large molecular surveys where costs are an important factor (**S13 Appendix**); and finally 5) It is compatible with volumes up to 25ml, potentially boosting sensitivity for even low abundant targets.

In fact, the inability to fully take advantage of large sample volumes may have previously led to an underestimation of the diagnostic utility of urine. As commercial kit-based methods are either incompatible or too expensive for use with large volumes, some studies have relied on centrifugation as a way to concentrate samples [20, 47–50]. While this will indeed increase nucleic acid yield, nearly all of it will originate from the pelleted cellular material, and not from the cell-free fraction. With this protocol, the hindrance of volume size has been removed and large volumes of whole urine can now be extracted in a high-throughput and cost-effective manner (**Fig 3, S5 Appendix**).

In fact, the 25ml upper limit of the current protocol simply reflects the maximum volume of the 50ml tubes used in this study. Therefore, higher volumes may be possible with larger storage vessels or by performing extractions in parallel. Nonetheless, it is notable that a 25ml sample of urine extracted with the current method yielded more nucleic acids than a 50μl dried blood spot sample extracted with Qiagen QIAamp (**S14 Appendix**). This ability to utilize larger volumes may have particular relevance for certain transrenal/cell-free DNAs that have been found to be particularly scarce in urine [28, 29]. It is therefore possible that the use of increased starting material, coupled with purification and amplification of both DNA and RNA with qRTPCR, may help to improve the detection of even very low abundant targets.

The present study also confirms that the cell-free nucleic acids in urine are small in nature (< 240bp), consistent with previous reports (**Fig 4**) [24–27, 42, 43]. It is important to note that the smallest amplicons (60-90bp) yielded the lowest overall qRTPCR Ct values, and that for amplicons > 180bp a specific loss in signal occurred for the cell-free nucleic acid fraction. Given these findings, when attempting to amplify cell-free targets with qRTPCR it is advisable to target highly expressed genes with the smallest amplicon possible, ideally with exonic primers that do not span introns (in order to amplify both DNA and RNA targets).

One short-coming of this study is that only a human target was used, and thus it will be essential to test how this method performs for the detection of actual human pathogens. In this regard, the use of RNA may yield an additional benefit—providing an ability to distinguish between latent and active infections.

The future of urine-based diagnostics is bright, with a number of biotechnology companies already attempting to commercialize tests for use in the clinic. As urine has already proven useful for detecting pathogens of global health importance, this sampling strategy may also hold great promise for epidemiological research in the developing world. This may be particularly relevant for Zika and Ebola, where urine-based testing is already an established diagnostic method [11–17]. In a similar fashion, urine-based testing of HPV may help improve screening rates or facilitate the large studies needed to evaluate the efficacy of HPV vaccination efforts. In this regard urine may prove as a more acceptable sampling strategy compared to the more invasive pap tests. The methods described here will hopefully be a step in the right direction to fully realize the diagnostic potential of urine. With further refinements, urine may eventually become a complementary or perhaps even superior sampling strategy for diagnosing a number of human diseases.

## Supporting information

S1 Appendix High-throughput nucleic acid extraction protocol with either home-made or commercial buffers for urine volumes up to 0.5ml. (DOCX)

S2 Appendix High-throughput nucleic acid extraction protocol with either home-made or commercial buffers for urine volumes up to 2.5ml. (DOCX)

S3 Appendix High-throughput nucleic acid extraction protocol with either home-made or commercial buffers for urine volumes up to 7.5ml. (DOCX)

S4 Appendix High-throughput nucleic acid extraction protocol with either home-made or commercial buffers for urine volumes up to 25ml. (DOCX)

S5 Appendix Schematic representation of the adapted protocol for urine volumes of 2.5ml, 7.5ml, and 25ml. Detailed protocols can be found in S2–S4 Appendices and list of consumables in S2 and S3 Tables.
(TIF)

S6 Appendix Primer, probe and cycling conditions used in this study for the human actin gene. (DOCX)

S7 Appendix Optimization of different lysis buffer conditions. Lysis buffer containing 3M guanidine thiocyanate (GuSCN) with 33% isopropanol (ISOH), 0.5% 2-mercaptoethanol at pH 6.5–6.0 used at 1:1 ratio with urine yields the lowest human *actin* Ct values as assessed by qRTPCR.
(DOCX)

S8 Appendix Testing of different commercially available 96-well DNA binding plates. Nunc DNA plates yield the lowest Ct values for human *actin* as assessed by qRTPCR.
(DOCX)

S9 Appendix Testing of different wash conditions. Addition of 2-mercaptoethanol (BM) or other additives to Wash 1 does not markedly improve purification efficiency.
(DOCX)

S10 Appendix Optimization of the current protocol for use with larger urine volumes. The appropriate amount of silica (see materials and method and S1 Appendix) and which DNA plates to use were derived empirically.
(DOCX)

S11 Appendix Addition of BSA (0.1-1mg/ml) to urine, but not to silica or lysis buffer, improves *actin* Ct values. (DOCX)

S12 Appendix Qiagen RLT-plus performs equivalently as the home-made lysis buffer for purification of nucleic acids from urine. (DOCX)

S13 Appendix The cost of the current method using home-made or RLT-plus buffers for varying volumes of urine. Included are costs for all consumables required for extraction per sample.
(DOCX)

S14 Appendix A 25ml sample of urine yields more nucleic acids than a 50μl dried blood spot sample extracted with Qiagen QIAamp. (DOCX)

S1 Table List of consumables and equipment needed for extraction of urine volumes up to 500μl. (XLS)

S2 Table List of consumables and equipment needed for extraction of urine volumes up to 2.5ml. (XLS)

S3 Table List of consumables and equipment needed for extraction of urine volumes up to 25ml. (XLS)
