# Supplementary material for: An efficient and cost-effective method for purification of small sized DNAs and RNAs from human urine
Source: PLoS One. 2019 Feb 5;14(2):e0210813. doi: 10.1371/journal.pone.0210813 (PMC6363378; doi:10.1371/journal.pone.0210813)
Supplement: S1 Appendix — (DOCX) [file pone.0210813.s001.docx]

**S1 Appendix. High-throughput nucleic acid extraction protocol with either home-made or commercial buffers for urine volumes up to 0.5ml.**

|  | **Homemade**  **Buffers** | **Commercial**  **Buffer Substitutes** |
| --- | --- | --- |
| **Lysis:** | 3M Guanidine thiocyanate  33% Isopropanol  4% Triton X100  50mM EDTA  20mM Trizma HCl pH 7.40  .5% 2-mercaptoethanol  pH 6.0-6.5 | Qiagen RLT-plus  33% Isopropanol  0.5% 2-mercaptoethanol |
| **Wash 1:** | Lysis buffer diluted 1:1 with water  (no 2-mercaptoethanol) | Lysis buffer diluted 1:1 with water  (no 2-mercaptoethanol) |
| **Wash 2:** | 25% Ethanol  25% Isopropanol  100mM Sodium chloride  10mM Trizma HCl pH 7.4 | 70% ethanol, 30% PBS |

NOTE: Lysis and Wash 1 contain guanidine thiocyanate which is toxic and **INCOMPATIBLE** with bleach

1. Transfer 500µl of urine sample to a 96 well deep well plate^*^
   1. Take proper precaution to prevent cross-contamination of samples (*ie* use of plate covers)
2. Add 500µl of lysis buffer (using a multichannel pipette)
3. Mix by pipetting up and down with multichannel pipette before transferring 750µl to a Nunc 96^#^ well DNA plate (Z688673, Sigma) sitting on top of a 2ml 96 well deep well collection plate
4. Spin 3,700RPM (2,250 g) for 1 minute
5. Pipette 500µl of Wash 1
6. Spin 3,700RPM (2,250 g) for 1 minute
7. Pipette 500µl of Wash 2
8. Spin 3,700RPM (2,250 g) for 2 minutes
9. Dry DNA plate at 56-65°C for 10 minutes
10. Place DNA plate on top of 0.5ml 96 well plate
11. Pipette 50µl of TE buffer pH 8.0
12. Spin 3,700RPM (2,250 g) for 2 minutes

^*^ A complete list of consumables and equipment can be found in S1 Table.

^#^ Omega EZ 96-well DNA plates (95030-226, VWR) are less expensive though display slightly reduced sensitivity
