# Supplementary material for: An efficient and cost-effective method for purification of small sized DNAs and RNAs from human urine
Source: PLoS One. 2019 Feb 5;14(2):e0210813. doi: 10.1371/journal.pone.0210813 (PMC6363378; doi:10.1371/journal.pone.0210813)
Supplement: S2 Appendix — (DOCX) [file pone.0210813.s002.docx]

**S2 Appendix. High-throughput nucleic acid extraction protocol with either home-made or commercial buffers for urine volumes up to 2.5ml.**

|  | **Homemade**  **Buffers** | **Commercial**  **Buffer Substitutes** |
| --- | --- | --- |
| **Lysis:** | 3M Guanidine thiocyanate  33% Isopropanol  4% Triton X100  50mM EDTA  20mM Trizma HCl pH 7.40  .5% 2-mercaptoethanol  pH 6.0-6.5 | Qiagen RLT-plus  33% Isopropanol  0.5% 2-mercaptoethanol |
| **Wash 1:** | Lysis buffer diluted 1:1 with water  (no 2-mercaptoethanol) | Lysis buffer diluted 1:1 with water  (no 2-mercaptoethanol) |
| **Wash 2:** | 25% Ethanol  25% Isopropanol  100mM Sodium chloride  10mM Trizma HCl pH 7.4 | 70% ethanol, 30% PBS |

NOTE: Lysis and Wash 1 contain guanidine thiocyanate which is toxic and **INCOMPATIBLE** with bleach

1. Before beginning, you must first prepare the **binding solution** of silicon dioxide (S5631, Sigma Aldrich). The silica particles range in size from 0.5-10µm, to preferentially retain the larger sized particles thoroughly mix 2.5 grams with 50ml of water, allow to settle for 2 hours, and discard the supernatant. Repeat 2 additional times. Resuspend the resulting pellet with 20ml of lysis buffer (**see table above**).
2. Transfer 2.5ml of urine to a 5ml tube^*^
3. Add 2.5ml of lysis buffer and 150µl of silica binding solution from step 1^#^ (vortex to resuspend silica before aliquoting)
4. Mix thoroughly
5. Spin 3,000 RPM (1,640 g) for 1 minute
6. Discard supernatant
7. Resuspend pellet with 500µl of Wash 1 and transfer to a Pall GHP .45µm 96-well size exclusion plate^@^ (89233-870, VWR) sitting on top of a 2ml 96 well deep well collection plate
8. Spin 3,700RPM (2,250 g) for 1 minute
9. Pipette 500µl of Wash 1 (using multichannel pipette)
10. Spin 3,700RPM (2,250 g) for 1 minute
11. Pipette 500µl of Wash 2
12. Spin 3,700RPM (2,250 g) for 2 minutes
13. Dry DNA plate at 56-65°C for 10 minutes
14. Place DNA plate on top of 0.5ml 96 well plate
15. Pipette 50µl of TE buffer pH 8.0
16. Spin 3,700RPM (2,250 g) for 2 minutes

^*^ A complete list of consumables and equipment can be found in S2 Table.

^#^ Silica can be added directly to lysis buffer if performing many extractions. Just make sure to mix thoroughly in order to resuspend the silica.

^@^ Pall Supor 1.2µm plates (97052-128, VWR) are less expensive and also compatible, though may display slightly reduced sensitivity
