# Supplementary figures and images for: An efficient and cost-effective method for purification of small sized DNAs and RNAs from human urine
Source: PLoS One. 2019 Feb 5;14(2):e0210813. doi: 10.1371/journal.pone.0210813 (PMC6363378; doi:10.1371/journal.pone.0210813)

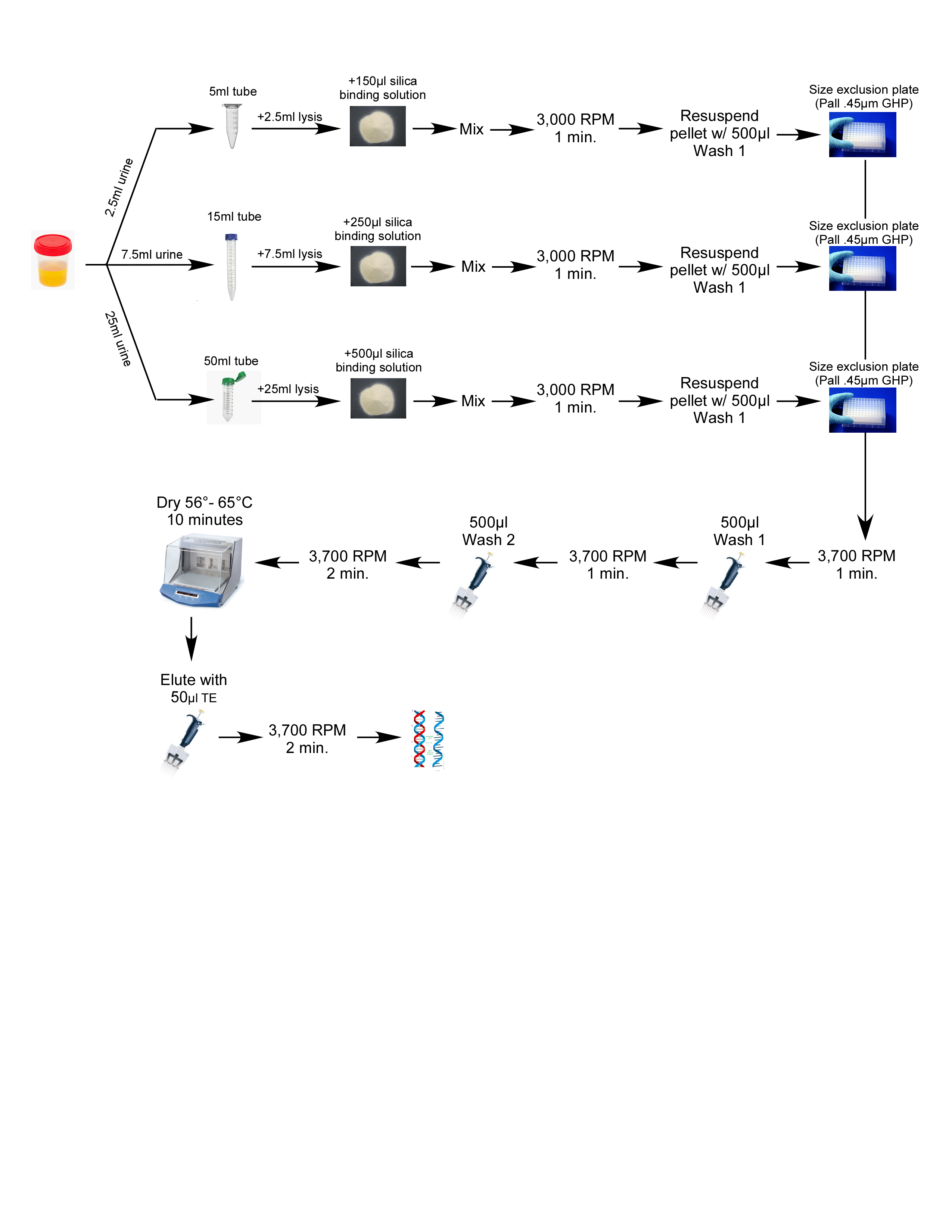

Supplement: S5 Appendix — Detailed protocols can be found in S2–S4 Appendices and list of consumables in S2 and S3 Tables. (TIF) [file pone.0210813.s005.tif]
