# Supplementary material for: An efficient and cost-effective method for purification of small sized DNAs and RNAs from human urine
Source: PLoS One. 2019 Feb 5;14(2):e0210813. doi: 10.1371/journal.pone.0210813 (PMC6363378; doi:10.1371/journal.pone.0210813)
Supplement: S6 Appendix — (DOCX) [file pone.0210813.s006.docx]

| Amplicon size (bp) | Primer/ probe | Sequence (5’-3’) | qPCR cycling conditions | qRTPCR Cycling conditions |
| --- | --- | --- | --- | --- |
| - | R | CTT AAT GTC ACG CAC GAT TTC C | 95°C 15 minutes  94°C 45 seconds*  60°C 75 seconds  *Repeat 44 times | 50°C 20 minutes (RT)  95°C 15 minutes  94°C 45 seconds*  60°C 75 seconds  *Repeat 44 times |
| 60 | F | ACC GAG CGC GGC TAC AG |  |  |
| 92 | F | GGG ACC TGA CTG ACT ACC TCAT G |  |  |
| 180 | F | GAC TCC GGT GAC GGG GTC |  |  |
| 241 | F | CGT TGC TAT CCA GGC TGT GC |  |  |
| 480 | F | TGG CTC GTG TGA CAA GGC |  |  |
| - | Probe | VIC-TTC ACC ACC ACG GCC GAG C-MGB |  |  |

**S6 Appendix. Primer, probe and cycling conditions used in this study for the human actin gene.**

VIC (life Technologies); MGB, minor-groove binder; qPCR, quantitative polymerase chain reaction; RT, reverse-transcription
