# Supplementary material for: An efficient and cost-effective method for purification of small sized DNAs and RNAs from human urine
Source: PLoS One. 2019 Feb 5;14(2):e0210813. doi: 10.1371/journal.pone.0210813 (PMC6363378; doi:10.1371/journal.pone.0210813)
Supplement: S7 Appendix — Lysis buffer containing 3M guanidine thiocyanate (GuSCN) with 33% isopropanol (ISOH), 0.5% 2-mercaptoethanol at pH 6.5–6.0 used at 1:1 ratio with urine yields the lowest human actin Ct values as assessed by qRTPCR. (DOCX) [file pone.0210813.s007.docx]

**S7 Appendix. Optimization of different lysis buffer conditions.** Lysis buffer containing 3M guanidine thiocyanate (GuSCN) with 33% isopropanol (ISOH), 0.5% 2-mercaptoethanol at pH 6.5-6.0 used at 1:1 ratio with urine yields the lowest human *actin* Ct values as assessed by qRTPCR.

|  | 3M GuSCN | | 4M GuSCN | | 6M GuSCN | |
| --- | --- | --- | --- | --- | --- | --- |
| ISOH: | 33% | 20% | 33% | 20% | 33% | 20% |
| Average Ct  (± SD) | 26.8  (±.2) | 27.5  (±.1) | 27.5  (±.1) | 27.5  (±.1) | 27.2  (±.1) | 27.2  (±.1) |

|  | 3M GuSCN 33.3% ISOH | | 3M GuSCN 20% ISOH | |
| --- | --- | --- | --- | --- |
| Lysis : Urine (volume) | 1:1 | 2:1 | 1:1 | 2:1 |
| Average Ct  (± SD) | 26.6  (±.2) | 27.3  (±.3) | 27.3  (±.4) | 28.2  (±.1) |

|  | 3M GuSCN + 33.3% ISOH | |
| --- | --- | --- |
| pH: | 6.5 | 6.0 |
| Average Ct  (± SD) | 28.6  (±.1) | 28.6  (±.1) |

|  | 3M GuSCN + 33.3% ISOH | | | |
| --- | --- | --- | --- | --- |
| 2-mercaptoethanol: | 0% | 0.1% | 0.33% | 1% |
| Average Ct  (± SD) | 34.5  (±.2) | 33.9  (±.8) | 32.8  (±.3) | 32.9  (±.3) |

|  | 3M GuSCN + 33.3% ISOH | | | |
| --- | --- | --- | --- | --- |
| 2-mercaptoethanol: | 0% | 0.25% | 0.5% | 1% |
| Average Ct  (± SD) | 26.2  (±.3) | 25.9  (±.3) | 24.9  (±.2) | 25.1  (±.2) |

GuSCN, guanidine thiocyanate; Ct, cycle threshold; SD, standard deviation; ISOH, isopropanol
