# Supplementary material for: An efficient and cost-effective method for purification of small sized DNAs and RNAs from human urine
Source: PLoS One. 2019 Feb 5;14(2):e0210813. doi: 10.1371/journal.pone.0210813 (PMC6363378; doi:10.1371/journal.pone.0210813)
Supplement: S8 Appendix — Nunc DNA plates yield the lowest Ct values for human actin as assessed by qRTPCR. (DOCX) [file pone.0210813.s008.docx]

**S8 Appendix. Testing of different commercially available 96-well DNA binding plates.** Nunc DNA plates yield the lowest Ct values for human *actin* as assessed by qRTPCR.

|  | 3M GuSCN + 33.3% ISOH | | |
| --- | --- | --- | --- |
| DNA Plate: | Omega EZ  (VWR) | Econospin  (Epoch Life sciences) | Pureplate  (E&K Scientific) |
| Average Ct  (± SD) | 25.8  (±.1) | 25.8  (±.4) | 25.9  (±.1) |

|  | 3M GuSCN + 33.3% ISOH | |
| --- | --- | --- |
| DNA plate: | Omega EZ  (VWR) | Nunc  (Sigma) |
| Average Ct  (± SD) | 26.9  (±.2) | 26.1  (±.2) |

GuSCN, guanidine thiocyanate; Ct, cycle threshold; SD, standard deviation; ISOH, isopropanol
