# Supplementary material for: An efficient and cost-effective method for purification of small sized DNAs and RNAs from human urine
Source: PLoS One. 2019 Feb 5;14(2):e0210813. doi: 10.1371/journal.pone.0210813 (PMC6363378; doi:10.1371/journal.pone.0210813)
Supplement: S9 Appendix — Addition of 2-mercaptoethanol (BM) or other additives to Wash 1 does not markedly improve purification efficiency. (DOCX) [file pone.0210813.s009.docx]

**S9 Appendix. Testing of different wash conditions.** Addition of 2-mercaptoethanol (BM) or other additives to Wash 1 does not markedly improve purification efficiency.

|  | Wash 1 | |
| --- | --- | --- |
| BM: | 0% | 0.5% |
| Average Ct  (± SD) | 29.7  (±.1) | 30.0  (±.2) |

|  | Additives | | | | | |
| --- | --- | --- | --- | --- | --- | --- |
| Wash 1: | - | 0.5% BM | .1mg/ml BSA | 1mg/ml BSA | .1mg/ml  α-casein | 1mg/ml  α-casein |
| Average Ct  (± SD) | 26.0  (±.1) | 26.1  (±.1) | 26.2  (±.3) | 25.9  (±.3) | 25.9  (±.1) | 25.7  (±.1) |

BM, 2-mercaptoethanol; SD, standard deviation; Ct, cycle threshold
