# Supplementary material for: An efficient and cost-effective method for purification of small sized DNAs and RNAs from human urine
Source: PLoS One. 2019 Feb 5;14(2):e0210813. doi: 10.1371/journal.pone.0210813 (PMC6363378; doi:10.1371/journal.pone.0210813)
Supplement: S10 Appendix — The appropriate amount of silica (see materials and method and S1 Appendix) and which DNA plates to use were derived empirically. (DOCX) [file pone.0210813.s010.docx]

**S10 Appendix. Optimization of the current protocol for use with larger urine volumes.** The appropriate amount of silica (see materials and method and S1 Appendix) and which DNA plates to use were derived empirically.

|  | 3M GuSCN + 33.3% ISOH (50ml) | | | |
| --- | --- | --- | --- | --- |
| Silica: | 125µl | 250µl | 500µl | 1ml |
| Average Ct  (± SD) | 27.3  (±.1) | 26.6  (±.3) | 26.5  (±.1) | 27.5  (±.1) |

|  | 3M GuSCN + 33.3% ISOH (50ml) | | |
| --- | --- | --- | --- |
| Size exclusion plate: | Pall GHP (.45µm) | Pall Supor  (1.2µm) | None (tube) |
| Average Ct  (± SD) | 25.4  (±.3) | 25.7  (±.2) | 27.1  (±.1) |

GuSCN, guanidine thiocyanate; SD, standard deviation; Ct, cycle threshold; ISOH, isopropanol
