# Supplementary material for: An efficient and cost-effective method for purification of small sized DNAs and RNAs from human urine
Source: PLoS One. 2019 Feb 5;14(2):e0210813. doi: 10.1371/journal.pone.0210813 (PMC6363378; doi:10.1371/journal.pone.0210813)
Supplement: S11 Appendix — (DOCX) [file pone.0210813.s011.docx]

**S11 Appendix. Addition of BSA (0.1-1mg/ml) to urine, but not to silica or lysis buffer, improves *actin* Ct values.**

|  | DNA plate (400µl urine) | | | |
| --- | --- | --- | --- | --- |
| Urine + [BSA]: | 0mg/ml | .1mg/ml | .33mg/ml | 1mg/ml |
| Average Ct  (± SD) | 28.7  (±.3) | 27.5  (±.2) | 27.5  (±.3) | 27.2  (±.1) |

|  | DNA plate (400µl urine) | | | |
| --- | --- | --- | --- | --- |
| Urine + [BSA]: | 0mg/ml | 1mg/ml | 3.3mg/ml | 10mg/ml |
| Average Ct  (± SD) | 28.4  (±1.1) | 27.6  (±.1) | 28.4  (±.1) | 29.3  (±.4) |

|  | GHP plate (2.5ml urine) | | | | Supor plate (2.5ml urine) | | | |
| --- | --- | --- | --- | --- | --- | --- | --- | --- |
| Urine + [BSA]: | 0mg/ml | 1mg/ml | 3.3mg/ml | 10mg/ml | 0mg/ml | 1mg/ml | 3.3mg/ml | 10mg/ml |
| Average Ct  (± SD) | 26.9  (±.3) | 25.9  (±.3) | 25.8  (±.2) | 26.2  (±.2) | 27.5  (±.1) | 26.7  (±.2) | 26.9  (±.3) | 27.2  (±.2) |

|  | GHP plate (2.5ml urine) | | | | Supor plate (2.5ml urine) | | | |
| --- | --- | --- | --- | --- | --- | --- | --- | --- |
| Silica + [BSA]: | 0mg/ml | 1mg/ml | 3.3mg/ml | 10mg/ml | 0mg/ml | 1mg/ml | 3.3mg/ml | 10mg/ml |
| Average Ct  (± SD) | 25.8  (±.3) | 25.9  (±.1) | 25.5  (±.5) | 25.9  (±.1) | 25.9  (±.2) | 26.2  (±.1) | 26.0  (±.1) | 25.8  (±.1) |

| 2.5ml urine | Additives | | | | |
| --- | --- | --- | --- | --- | --- |
| Lysis buffer: | - | BSA^*^ | α-casein^@^ | - | - |
| Silica: | - | - | - | BSA^*^ | α-casein^@^ |
| Average Ct  (± SD) | 26.4  (±.3) | 26.9  (±.1) | 26.6 (±.1) | 27.3  (±.2) | 27.4  (±.1) |

| 25ml urine | Additives | | | |
| --- | --- | --- | --- | --- |
| Lysis buffer: | - | BSA^*^ | - | BSA^*^ |
| Silica: | - | - | α-casein^@^ | α-casein^@^ |
| Average Ct  (± SD) | 24.5  (±.2) | 25.4  (±.3) | 24.3  (±.3) | 24.5  (±.2) |

^*^ BSA, 1mg/ml; ^@^ α-casein, 0.1mg/ml; SD standard deviation; Ct, cycle threshold
