# Supplementary material for: An efficient and cost-effective method for purification of small sized DNAs and RNAs from human urine
Source: PLoS One. 2019 Feb 5;14(2):e0210813. doi: 10.1371/journal.pone.0210813 (PMC6363378; doi:10.1371/journal.pone.0210813)
Supplement: S12 Appendix — (DOCX) [file pone.0210813.s012.docx]

**S12 Appendix. Qiagen RLT-plus performs equivalently as the home-made lysis buffer for purification of nucleic acids from urine.**

| Lysis buffer: | 3M GuSCN + 33.3% ISOH | RLT-plus + 33.3% ISOH |
| --- | --- | --- |
| Average Ct  (± SD) | 29.8  (±.1) | 29.7  (±.2) |

1:1 urine to lysis buffer; GuSCN, guanidine thiocyanate; ISOH, isopropanol; Ct, cycle threshold; SD, standard deviation
