# Supplementary material for: An efficient and cost-effective method for purification of small sized DNAs and RNAs from human urine
Source: PLoS One. 2019 Feb 5;14(2):e0210813. doi: 10.1371/journal.pone.0210813 (PMC6363378; doi:10.1371/journal.pone.0210813)
Supplement: S13 Appendix — Included are costs for all consumables required for extraction per sample. (DOCX) [file pone.0210813.s013.docx]

**S13 Appendix. The cost of the current method using home-made or RLT-plus buffers for varying volumes of urine.** Included are costs for all consumables required for extraction per sample.

| Urine volume: | 400µl | | | 2.5ml | | 25ml | |
| --- | --- | --- | --- | --- | --- | --- | --- |
| Method: | Home-made | RLT-plus buffers | Qiagen QIAamp | Home-made | RLT-plus buffers | Home-made | RLT-plus buffers |
| Cost per sample: | $1.75 | $2.01 | $5.00 | $2.10 | $3.08 | $5.44 | $14.57 |
