# Supplementary material for: An efficient and cost-effective method for purification of small sized DNAs and RNAs from human urine
Source: PLoS One. 2019 Feb 5;14(2):e0210813. doi: 10.1371/journal.pone.0210813 (PMC6363378; doi:10.1371/journal.pone.0210813)
Supplement: S14 Appendix — (DOCX) [file pone.0210813.s014.docx]

**S14 Appendix. A 25ml sample of urine yields more nucleic acids than a 50µl dried blood spot sample extracted with Qiagen QIAamp.**

|  | Urine (25ml) | | Dried blood spot (50µl) | Whole blood (50µl) |
| --- | --- | --- | --- | --- |
|  | Male | Female |  |  |
| Average Ct  (± SD) | 24.4  (±.1) | 20.2  (±.1) | 27.2  (±.5) | 22.2  (±.2) |

Ct, cycle threshold; SD, standard deviation
